# Complete genome sequence of *Sanguibacter keddieii* type strain (ST-74^T^)

**DOI:** 10.4056/sigs.16197

**Published:** 2009-09-24

**Authors:** Natalia Ivanova, Johannes Sikorski, David Sims, Thomas Brettin, John C. Detter, Cliff Han, Alla Lapidus, Alex Copeland, Tijana Glavina Del Rio, Matt Nolan, Feng Chen, Susan Lucas, Hope Tice, Jan-Fang Cheng, David Bruce, Lynne Goodwin, Sam Pitluck, Amrita Pati, Konstantinos Mavromatis, Amy Chen, Krishna Palaniappan, Patrik D’haeseleer, Patrick Chain, Jim Bristow, Jonathan A. Eisen, Victor Markowitz, Philip Hugenholtz, Markus Göker, Rüdiger Pukall, Hans-Peter Klenk, Nikos C. Kyrpides

**Affiliations:** 1 DOE Joint Genome Institute, Walnut Creek, California, USA; 2 DSMZ - German Collection of Microorganisms and Cell Cultures GmbH, Braunschweig, Germany; 3 Los Alamos National Laboratory, Bioscience Division, Los Alamos, New Mexico, USA; 4 Biological Data Management and Technology Center, Lawrence Berkeley National Laboratory, Berkeley, California, USA; 5 Lawrence Livermore National Laboratory, Livermore, California, USA; 6 University of California Davis Genome Center, Davis, California, USA

**Keywords:** blood isolate, aerobic, facultative anaerobic, *Sanguibacteraceae*, *Micrococcineae*

## Abstract

*Sanguibacter keddieii* is the type species of the genus *Sanguibacter*, the only genus within the family of *Sanguibacteraceae*. Phylogenetically, this family is located in the neighborhood of the genus *Oerskovia* and the family *Cellulomonadaceae* within the actinobacterial suborder *Micrococcineae*. The strain described in this report was isolated from blood of apparently healthy cows. Here we describe the features of this organism, together with the complete genome sequence, and annotation. This is the first complete genome sequence of a member of the family *Sanguibacteraceae,* and the 4,253,413 bp long single replicon genome with its 3735 protein-coding and 70 RNA genes is part of the *** G****enomic* *** E****ncyclopedia of* *** B****acteria and* *** A****rchaea * project.

## Introduction

Strain ST-74^T^ (= DSM 10542 = ATCC 51767 = JCM 11429 = NCIMB 703025) is the type strain of *Sanguibacter keddieii*, which is the type species of the genus *Sanguibacter* [1]. *S. keddieii* strain ST-74^T^ was isolated in 1995 by Fernandez-Garayzabal *et al*. from the blood of apparently healthy dairy cows in Spain [1] as the first member of the genus *Sanguibacter* and the family of *Sanguibacteraceae* [2]. On the basis of 16S rRNA sequence phylogeny, the small (six species, one genus) family *Sanguibacteraceae* is located in the neighborhood to the genus *Oerskovia* [3], now part of the *Cellulomonadaceae* [2], as well as the *Promicromonosporaceae.* Here we present a summary classification and a set of features for *S. keddieii* ST-74^T^ together with the description of the complete genomic sequencing and annotation.

## Classification and features

Like strain ST-74^T^, two more type strains from the genus *Sanguibacter* (*S. suarezii* ST-26^T^ [1], and *S. inulinus* [4]) have been isolated from blood of cows. The type strains of the other *Sanguibacter* species have been isolated from coastal sediment in the Eastern China Sea [5], from surface soil of a ginseng field in South Korea [6], from alpine subnival plants (DQ339590), and from a sea sand sample collected on the Weaver Peninsula on King George Island, Antarctica [7], which may suggest a global ecological versatility of this genus. Only two related but yet uncultivated phylotypes with more than 98.5% 16S rRNA sequence identity were reported from the gastrointestinal tract of pigs (AF371710), and from glacial meltwater at 6,350 m on Mount Everest (EU584523), and no significant matches with any 16S rRNA sequences from environmental genomic samples and surveys are reported at the NCBI BLAST server (March 2009).

**Figure 1:**
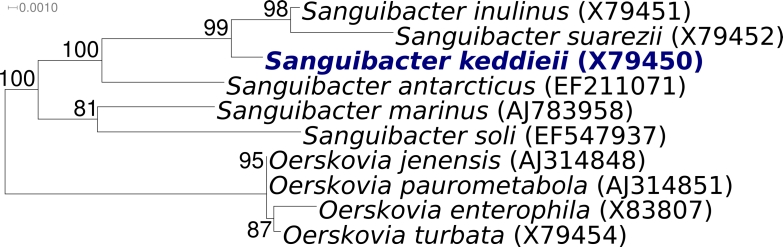
Phylogenetic tree of *S. keddieii* strain ST-74^T^ with all type strains of the family *Sanguibacteraceae*, inferred from 1,468 aligned characters [8] of the 16S rRNA sequence under the maximum likelihood criterion [9,10]. The tree was rooted with the type strains from the neighbor genus *Oerskovia*. The branches are scaled in terms of the expected number of substitutions per site. Numbers above branches are support values from 1,000 bootstrap replicates if larger than 60%. Strains with a genome sequencing project registered in GOLD [11] are printed in blue; published genomes in bold.

Figure 1 shows the phylogenetic neighborhood of *S. keddieii* strain ST-74^T^ in a 16S rRNA based tree. Analysis of the four 16S rRNA gene sequences in the genome of strain ST-74^T^ indicated that the genes differ by up to two nucleotides from each other, with two of the copies being identical with the previously published 16S rRNA sequence generated from DSM 10542 (X79450).

**Figure 2:**
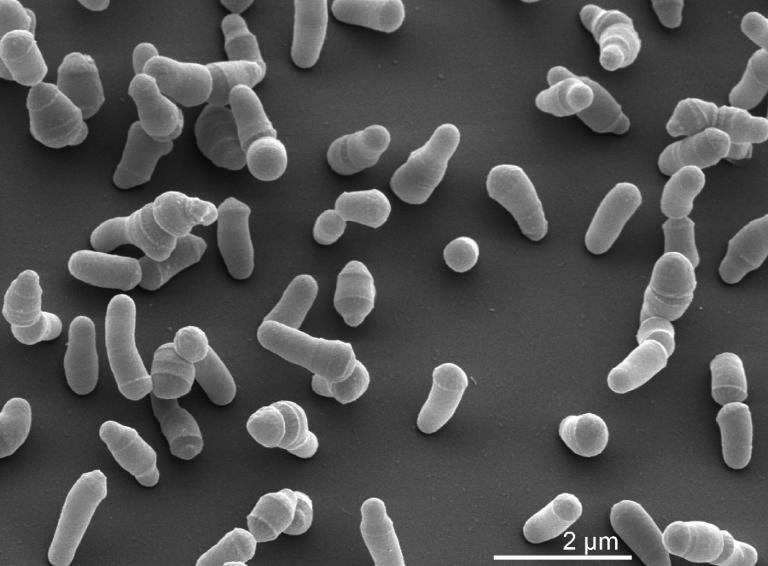
Scanning electron micrograph of *S. keddieii* ST-74 ^T^ (Manfred Rohde, Helmholtz Centre for Infection Biology, Braunschweig)

*S. keddieii* ST-74^T^ cells are facultatively anaerobic, Gram-positive, short, irregular shaped motile rods [1] (Table 1 and Figure 2). The colonies on tryptose soy agar (TSA, Difco) are circular, convex, with entire edges and yellow in color. Strain ST-74^T^ is Voges-Proskauer negative and does not reduce nitrate. Casein and gelatin are hydrolyzed. Cellulose and Tween 80 are not hydrolyzed. Acid is produced from a broad range of substrates: α-methyl-D-mannoside, α-methyl-D-glucoside, N-acetylglucosamine, amygdalin, rhamnose, D-rafinose, glycerol, L-arabinose, ribose, D-xylose, β-methyl-xyloside, galactose, glucose, fructose, D-mannose, rhamnose, arbutin, sorbitol, salicin, cellobiose, maltose, lactose, melibiose, sucrose, trehalose, raffinose, glycogen, β-gentibiose, turanose and lyxose [1]. The optimum growth temperature of strain ST-74^T^ is 25-30°C [1]; it grows at 35°C on agar [7] but not at 42°C [1].

**Table 1:** Classification and general features of *S. keddieii* ST-74 ^T^ according to the MIGS recommendations [12]

**MIGS ID**	**Property**	**Term**	**Evidence code**
	Current classification	Domain *Bacteria*	TAS [13]
		Phylum *Actinobacteria*	TAS [14]
		Class *Actinobacteria*	TAS [2]
		Order *Actinomycetales*	TAS [2]
		Family *Sanguibacteraceae*	TAS [15]
		Genus *Sanguibacter*	TAS [1]
		Species *Sanguibacter keddieii*	TAS [1]
		Type strain ST-74	
	Gram stain	positive	TAS [1]
	Cell shape	short, irregular rods	TAS [1]
	Motility	motile	TAS [1]
	Sporulation	not reported	
	Temperature range	mesophilic	TAS [1]
	Optimum temperature	25-30°C	TAS [1]
	Salinity	not reported	
MIGS-22	Oxygen requirement	primarily aerobe; facultatively anaerobic; no nitrate reduction	TAS [1]
	Carbon source	broad variety of sugars	TAS [1]
	Energy source	carbohydrates	NAS
MIGS-6	Habitat	animal blood	TAS [1]
MIGS-15	Biotic relationship	free living	NAS
MIGS-14	Pathogenicity	none	NAS
	Biosafety level	2	TAS [16]
	Isolation	blood of apparently healthy cow	TAS [1]
MIGS-4	Geographic location	Spain	NAS
MIGS-5	Sample collection time	before 1995	TAS [1]
MIGS-4.1 MIGS-4.2	Latitude , Longitude	not reported	
MIGS-4.3	Depth	not reported	
MIGS-4.4	Altitude	not reported	

Evidence codes - IDA: Inferred from Direct Assay (first time in publication); TAS: Traceable Author Statement (i.e., a direct report exists in the literature); NAS: Non-traceable Author Statement (i.e., not directly observed for the living, isolated sample, but based on a generally accepted property for the species, or anecdotal evidence). These evidence codes are from the Gene Ontology project [17]. If the evidence code is IDA, then the property was directly observed for a living isolate by one of the authors or another expert mentioned in the acknowledgements.

Little is known about the chemotaxonomy of strain ST-74^T^. The major cellular fatty acids are saturated straight chain and branched-chain forms. In strain ST-74^T^, the straight chain fatty acids 16:0 (53.3%), 18:0 (10.1%), 14:0 (5.8%) predominate over lower amounts of branched chain anteiso-15:0 (11.4%) and iso-16:0 (5.4%) fatty acids. This is in contrast to other species in the genus *Sanguibacter* and in the neighboring *Oerskovia* and *Cellulomonadaceae*, where branched chain fatty acids are predominant [18]. Only traces of unsaturated acids, anteiso-15:1 (1.6%), and no mycolic acids were detected [1], as in the neighboring taxa *Oerskovia* and other members of *Cellulomonadaceae*. The murein of *S. keddieii* contains L-Lys-Ser-D-Glu, variation A4α [1], strikingly different from members of the genus *Oerskovia* and other members of the family *Cellulomonadaceae* [1]. Menaquinones are the sole respiratory lipoquinones present, with a partially saturated menaquinone containing nine-isoprene subunits MK-9(H_4_) predominating [1]. The location of the points of unsaturation are in the second and third isoprene units, adjacent to the napthoquinone nucleus (MK-9 (II, III-H_4_), in *O. turbata*. The phospholipid composition has not been reported, but phosphatidylglycerol, diphosphatidylglycerol, phosphatidylinositol, together with phosphoglycolipids have been reported in members of the neighboring taxa *Oerskovia* and other members of the *Cellulomonadaceae* [18].

## Genome sequencing and annotation

### Genome project history

This organism was selected for sequencing on the basis of its phylogenetic position, and is part of the *** G****enomic* *** E****ncyclopedia of* *** B****acteria and* *** A****rchaea * project. The genome project is deposited in the Genome OnLine Database [11] and the complete genome sequence in GenBank. Sequencing, finishing and annotation were performed by the DOE Joint Genome Institute (JGI). A summary of the project information is shown in Table 2.

**Table 2:** Genome sequencing project information

MIGS ID	Property	Term
MIGS-31	Finishing quality	Finished
MIGS-28	Libraries used	Three genomic libraries: two Sanger libraries - 8 kb pMCL200 and fosmid pcc1Fos –
		and one 454 pyrosequence standard library
MIGS-29	Sequencing platforms	ABI3730, 454 GS FLX
MIGS-31.2	Sequencing coverage	10.4× Sanger; 20× pyrosequence
MIGS-30	Assemblers	Newbler version 1.1.02.15, phrap
MIGS-32	Gene calling method	Genemark 4.6b, tRNAScan-SE-1.23, infernal 0.81
	INSDC / Genbank ID	19711
	Genbank Date of Release	August 30, 2009
	GOLD ID	Gc01087
	NCBI Project ID	19711
	Database: IMG-GEBA	2500901759
MIGS-13	Source material identifier	DSM 10542
	Project relevance	Tree of Life, GEBA

### Growth conditions and DNA isolation

*S. keddieii* ST-74^T^, DSM10542, was grown in DSMZ medium 92 (3% trypticase soy broth, 0.3% yeast extract) [19] at 30°C. DNA was isolated from 1-1.5 g of cell paste using Qiagen Genomic 500 DNA Kit (Qiagen, Hilden, Germany) following the manufacturer’s protocol, but with extended (one hour) incubation at 37°C as described in Wu *et al.* [20

### Genome sequencing and assembly

The genome was sequenced using a combination of Sanger and 454 sequencing platforms. All general aspects of library construction and sequencing can be at found the JGI website (http://www.jgi.doe.gov). 454 Pyrosequencing reads were assembled using the Newbler assembler (Version 1.1.02.15, Roche). Large Newbler contigs were broken into 4,746 overlapping fragments of 1,000 bp and entered into assembly as pseudo-reads. The sequences were assigned quality scores based on Newbler consensus q-scores with modifications to account for overlap redundancy and to adjust inflated q-scores. A hybrid 454/Sanger assembly was made using the parallel phrap assembler (High Performance Software, LLC). Possible mis-assemblies were corrected with Dupfinisher [21] or transposon bombing of bridging clones (Epicentre Biotechnologies, Madison, WI). Gaps between contigs were closed by editing in Consed, custom primer walking, or PCR amplification. A total of 2,397 Sanger finishing reads were produced to close gaps, to resolve repetitive regions, and to raise the quality of the finished sequence. The error rate of the completed genome sequence was less than 1 in 100,000. Together all sequence types provided 30.4× coverage of the genome.

### Genome annotation

Genes were identified using GeneMark [22] as part of the genome annotation pipeline in the Integrated Microbial Genomes Expert Review (IMG-ER) system [23], followed by a round of manual curation using the JGI GenePRIMP pipeline (http://geneprimp.jgi-psf.org) [24]. The predicted coding sequences (CDS)s were translated and used to search the National Center for Biotechnology Information (NCBI) nonredundant database, UniProt, TIGRFam, Pfam, PRIAM, KEGG, COG, and InterPro databases. The tRNAScanSE tool [25] was used to find tRNA genes, whereas ribosomal RNAs were found by using the tool RNAmmer [26]. Other non coding RNAs were identified by searching the genome for the Rfam profiles using INFERNAL (v0.81) [27]. Additional gene prediction analysis and manual functional annotation was performed within the Integrated Microbial Genomes (IMG) platform [28].

### Metabolic network analysis

The metabolic Pathway/Genome Database (PGDB) was generated computationally using Pathway Tools software version 12.5 [29] and MetaCyc version 12.5 [30], based on annotated EC numbers and a customized enzyme name mapping file. This metabolic map has undergone no subsequent manual curation and may contain errors, similar to a Tier 3 BioCyc PGDB [31].

## Genome properties

**Figure 3:**
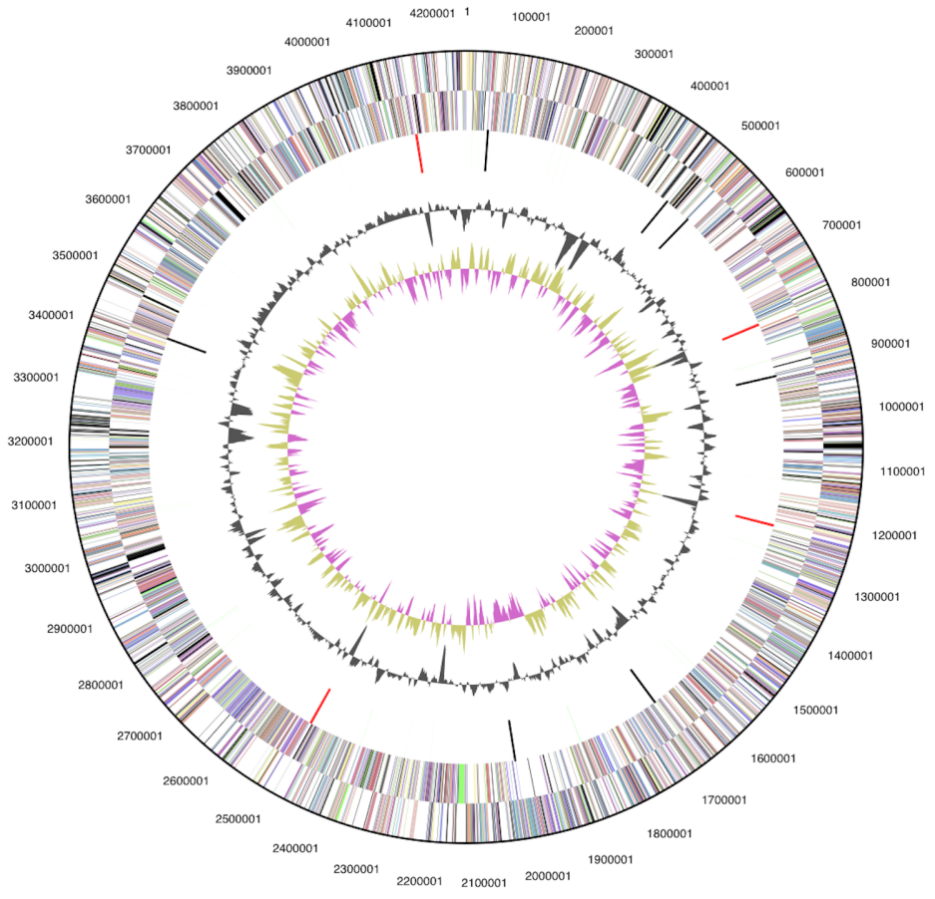
**Graphical circular map of the genome.** From outside to the center: Genes on forward strand (color by COG categories), Genes on reverse strand (color by COG categories), RNA genes (tRNAs green, rRNAs red, other RNAs black), GC content, GC skew

**Figure 4:**
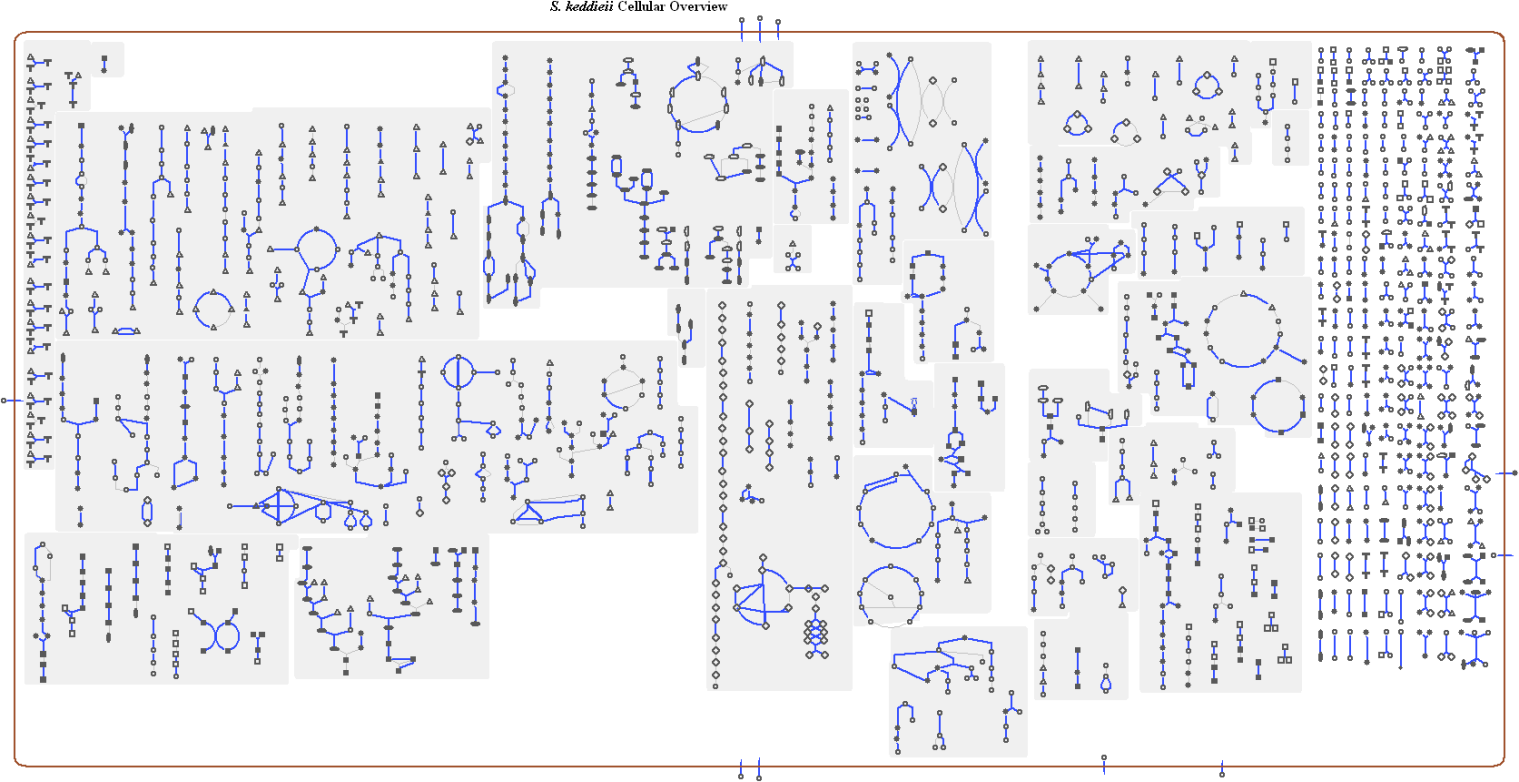
Schematic cellular overview diagram of all pathways of the *S. keddieii* ST-74^T^ metabolism. Nodes represent metabolites, with shape indicating class of metabolite (see key to right). Lines represent reactions.

The genome is 4,253,413 bp long and comprises one main circular chromosome with a 71.9% GC content (Table 3 and Figure 3). Of the 3,805 genes predicted, 3,735 were protein coding genes, and 70 RNAs. In addition, 25 pseudogenes were identified. The majority of the protein-coding genes (74.4%) were assigned with a putative function, while those remaining were annotated as hypothetical proteins. The properties and the statistics of the genome are summarized in Table 3. The distribution of genes into COGs functional categories is presented in Table 4. A cellular overview diagram is presented in Figure 4, followed by a summary of metabolic network statistics shown in Table 5.

**Table 3:** Genome Statistics

**Attribute**	**Value**	**% of Total**
Genome size (bp)	4,253,413	100.00%
DNA Coding region (bp)	3,872,139	91.04%
DNA G+C content (bp)	3,057,630	71.89%
Number of replicons	1	
Extrachromosomal elements	0	
Total genes	3,805	100.00%
RNA genes	70	1.84%
rRNA operons	4	
Protein-coding genes	3,735	98.16%
Pseudo genes	25	0.66%
Genes with function prediction	2,832	74.43%
Genes in paralog clusters	501	13.17%
Genes assigned to COGs	2,706	71.12%
Genes assigned Pfam domains	2,785	73.19%
Genes with signal peptides	912	23.97%
Genes with transmembrane helices	993	26.10%
CRISPR repeats	0	

**Table 4:** Number of genes associated with the general COG functional categories

**Code**	**Value**	**% age**	**Description**
J	166	5.0	Translation
A	1	0.0	RNA processing and modification
K	317	10.0	Transcription
L	120	4.0	Replication, recombination and repair
B	1	0.0	Chromatin structure and dynamics
D	25	1.0	Cell cycle control, mitosis and meiosis
Y	0	0.0	Nuclear structure
V	69	2.0	Defense mechanisms
T	173	6.0	Signal transduction mechanisms
M	134	4.0	Cell wall/membrane biogenesis
N	55	2.0	Cell motility
Z	3	0.0	Cytoskeleton
W	0	0.0	Extracellular structures
U	41	1.0	Intracellular trafficking and secretion
O	84	3.0	Posttranslational modification, protein turnover, chaperones
C	174	6.0	Energy production and conversion
G	354	12.0	Carbohydrate transport and metabolism
E	237	8.0	Amino acid transport and metabolism
F	77	3.0	Nucleotide transport and metabolism
H	119	4.0	Coenzyme transport and metabolism
I	80	3.0	Lipid transport and metabolism
P	199	7.0	Inorganic ion transport and metabolism
Q	43	1.0	Secondary metabolites biosynthesis, transport and catabolism
R	362	12.0	General function prediction only
S	213	7.0	Function unknown
-	1029	27.5	Not in COGs

**Table 5:** Metabolic Network Statistics

**Attribute**	**Value**
Total genes	3,805
Enzymes	714
Enzymatic reactions	935
Metabolic pathways	205
Metabolites	676
